# Superb microvascular imaging for detecting carpal tunnel syndrome compared with power Doppler ultrasonography: A protocol for systematic review and meta-analysis: Retraction

**DOI:** 10.1097/MD.0000000000042735

**Published:** 2025-06-06

The article, “Superb microvascular imaging for detecting carpal tunnel syndrome compared with power Doppler ultrasonography: A protocol for systematic review and meta-analysis”,^1^ which appears in Volume 100, Issue 9 of *Medicine*, is being retracted over concerns with the methodology. After publication, concerns were raised over references to the “Begger test,” which does not exist in the literature and is indicative of possible fraud. Upon further investigation, authorship irregularities were also discovered in our submission system. As a result, the Publisher no longer has confidence in the integrity of the content or the authorship.
